# Telomerase structure^☆^

**DOI:** 10.1016/j.sbi.2014.02.003

**Published:** 2014-04

**Authors:** Sara Sandin, Daniela Rhodes

**Affiliations:** 1 School of Biological Sciences, Nanyang Technological University, 60 Nanyang Drive, Singapore 637551, Singapore; 2 School of Biological Sciences and LKC Medicine, Proteos, 61 Biopolis Drive, Singapore 138673, Singapore

## Abstract

The telomerase reverse transcriptase has an essential role in telomere maintenance and in cancer biology. Progress during the last year has revealed the three-dimensional architecture of both human and ciliate telomerase at about 25 Å resolution, obtained using single particle electron microscopy (EM). The structural analysis of the two holoenzyme complexes isolated from cells shows that whilst the ciliate telomerase is monomeric, the human telomerase is dimeric and only functional as a dimer. We critically discuss the approaches taken to assign the location of protein and RNA subunits, as well as fitting the crystal structure of the catalytic protein subunit in the medium resolution EM density maps. Comparison of the two structural interpretations reveals not only a common RNA/reverse transcriptase core, but also significant differences due to different RNA subunit size and protein composition. These differences suggest that the oligomeric state and subunit composition of telomerase in evolutionary distant organism have evolved.

## Highlights

• First of telomerase architecture.
• Human telomerase functions as a dimer.
• Conserved RNA/reverse transcriptase core.

**Current Opinion in Structural Biology** 2014, **25**:104–110

This review comes from a themed issue on **Macromolecular machines**

Edited by **Karl-Peter Hopfner** and **Tom Smith**

For a complete overview see the Issue and the Editorial

Available online 2nd April 2014

0959-440X/$ – see front matter, Crown Copyright © 2014 Published by Elsevier Ltd. All rights reserved.

**http://dx.doi.org/10.1016/j.sbi.2014.02.003**

## Introduction

The telomerase-based mechanism for telomere maintenance of linear chromosomes is conserved in most eukaryotes (reviewed in [1,2]). Telomeres are the protein/DNA complexes that cap the ends of eukaryotic chromosomes and maintenance of their length is essential for genomic stability and cell viability (reviewed in [3]). Telomere shortening correlates with cellular aging and the majority cancer cells depend on the activation of telomerase to gain proliferative immortality. Stem and progenitor cells also express low levels of telomerase (reviewed in [4]).

Although the pioneers of telomere biology recognized over half a century ago that ends of eukaryotic chromosomes have a crucial function and that there is an end-replication problem requiring a specialized mechanism for maintaining telomere length (reviewed in [2]), it was the discovery in 1985 by Blackburn and Greider [5] of telomerase activity in extracts from the ciliate *Tetrahymena* that opened up the field to the molecular characterization of the telomerase enzyme. In the late 1980s, studies in ciliates lead to the identification of the RNA subunit TER [6] that encodes the template region for telomeric repeat synthesis and functions as a reverse transcriptase [6,7]. Not until 1997 was the gene for the telomerase catalytic subunit TERT identified when biochemical analysis in ciliates [8] and genetic approaches in yeast [9] came together [10]. These key discoveries then permitted the identification corresponding telomerase subunit genes in other species such as humans (reviewed in [2]). This in turn enabled studies on the role of telomerase in cell immortalisation [11] and paved the way for the overexpression of telomerase subunits for structural analyses.

Subsequent studies showed that TER and TERT together form a tight complex that is sufficient for telomeric DNA repeat synthesis *in vitro* [12,13]. However, species-specific accessory proteins implicated in telomerase assembly, activity and localization have been found associated with the telomerase holoenzyme isolated from vertebrates, yeast (reviewed in [2]) and ciliates [14]. Telomerase is a processive enzyme, and therefore likely to exist in multiple conformational states to accomplish telomeric DNA repeat addition. In the first step of DNA synthesis, the 3′ end of the G-overhang is positioned in the active site of TERT aligning on the RNA template in TER through base pair formation. In a second elongation step, nucleotide addition takes place to synthesise the telomeric DNA repeat. In a third step the telomerase translocates to restart telomeric repeat synthesis (reviewed in [1,2]).

In this review we will focus on recent progress in determining the 3D architecture, using single particle EM, of two full-length telomerase complexes isolated from mammalian cells [15^••^] and the ciliate *Tetrahymena* [16^••^]. We discuss the two low-resolution telomerase structures together with higher-resolution structural information on the TERT subunit and TER fragments. In addition, we highlight similarities and differences between the two structures.

## Telomerase recruitment

**Figure 1:**
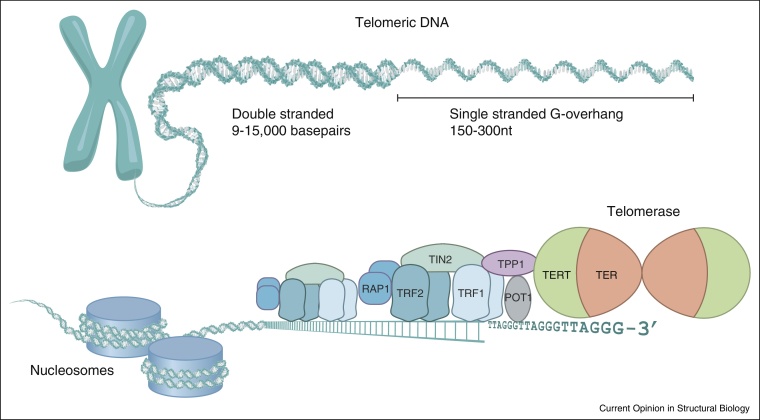
Human telomere structure and telomerase recruitment. Telomeric DNA consists of arrays of the TTAGGG telomeric repeat, forming a long region of double stranded DNA terminating in the single stranded G-rich overhang. In addition to being packaged by histones into chromatin, telomeric DNA is bound by the sheltering complex consisting of six telomere binding proteins: TRF1, TRF2, Rap1, TIN2, TPP1 and POT1. TRF1 and TRF2 bind sequence specifically as homodimers to double-stranded telomeric DNA, TIN2 forms a bridging interaction with TRF1 and TRF2 and TPP1. POT1 and TPP1 are localized to the G-overhang via the binding of POT1 to the single-stranded G-overhang. Telomerase is recruited to the tip of telomeres through the interaction of TPP1 with the N-terminal domain of the telomerase catalytic subunit TERT and through base pairing between the template region in the telomerase RNA subunit TER and the G-overhang.

Telomeres are the substrate for telomerase and hence their structure is involved in regulating DNA replication and telomerase recruitment [17]. Telomeric DNA from distantly related eukaryotes consists of tandem arrays of a conserved G-rich repeat (such as TTAGGG in humans) that protrudes from the telomeric end forming a single-stranded G-overhang (Figure 1). Telomere length is species-specific. The telomeres of macronuclear chromosomes in the ciliate *Tetrahymena thermophila* are short and consist of about 300 bp of telomeric DNA packaged into a non-nucleosomal complex [18]. In contrast, human telomeres are long. They consist of 1000 ds of bp of telomeric DNA that in addition to being packaged into nucleosomes, are bound by sequence-specific DNA binding proteins to form the telomeric capping structure called shelterin [3]. Structural determinations have revealed how telomeric proteins such as human TRF1 and TRF2 as well as POT1 recognize telomeric DNA [19–22,23^•^]. The G-overhang, which needs to be accessible for telomeric repeat addition by the telomerase, is in human telomeres bound by POT1 and TPP1. Importantly, the N-terminal OB (oligonucleotide/oligosaccharide binding)-fold of TPP1 was recently shown to control telomere maintenance by recruiting telomerase to the chromosome ends through a direct interaction [24^••^,25^••^,26,27]. This is consistent with an earlier observation from ciliates showing that the functional homologues of POT1 and TPP1, TEBPα and TEBPβ regulate telomerase recruitment through the cell-cycle dependent phosphorylation of TEBPβ [28]. The G-overhang binding yeast Cdc13 also functions in telomerase recruitment. TIN2, through its interaction with TPP1 as well as both TRF1 and TRF2 provides a bridge, linking single-strand and double-strand binding proteins in the shelterin complex (Figure 1) (reviewed in [3,26]). The interaction between shelterin components and telomerase must be transient since neither POT1 nor TPP1 co-purify with human telomerase [15^••^].

## High-resolution structures of TER and TERT subdomains

**Figure 2:**
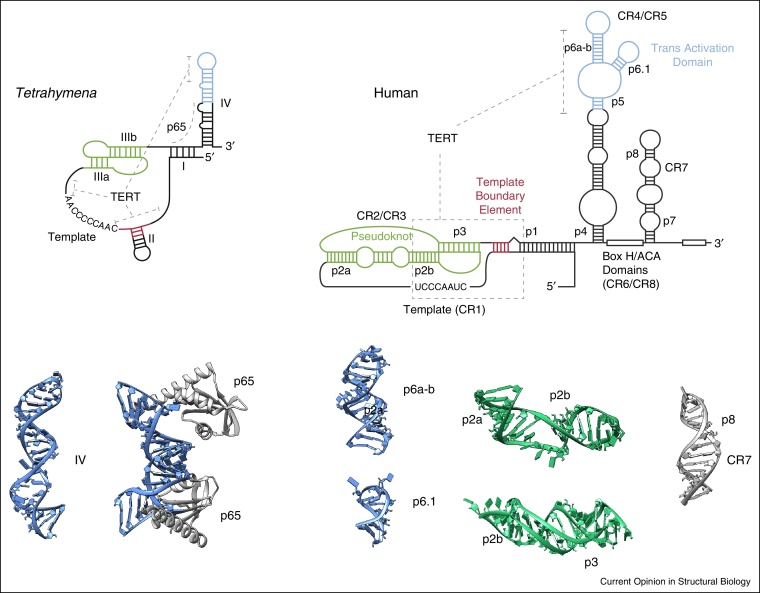
Domain structure of the telomerase RNA subunit TER. Secondary structures of the *Tetrahymena* and human telomerase TER subunits in which regions corresponding to the pseudo-knot (green), template boundary element (red), transactivation domain (blue) are highlighted. The interaction of TERT with TER domains is indicated (grey dotted line). The NMR structures of TER fragments are from the protein data bank and coloured according to the secondary structures: IV (pdb number: 2FEY), IV-p65 C-terminal domain (pdb number: 4ERD), p6a-b (pdb number: 2Z31), p6.1 (pdb number: 1OQ0), p2a/p2b (pdb number: 2LE3), p2b/p3 (pdb number: 2K96) and p8/CR7 (pdb number: 2QH2).

The RNA subunit TER is very divergent and ranges in length from about 150 nucleotides in ciliates to 450 in vertebrates to 1300 nucleotides in several yeasts. Despite these differences, phylogenetically derived secondary structure predictions revealed that all TER subunits contain two conserved structural elements: the catalytically essential pseudoknot-template core domain and a stem-loop element called CR4-CR5 in vertebrates (reviewed in [1]). These two conserved TER structural elements have been shown to interact directly with TERT [29]. The 3D structural information on TER is limited to isolated fragments [30,31] (Figure 2). These structures primarily confirm the secondary structure predictions. The crystal structure of the C-terminal domain of p65 in complex with stem IV reveals the details of RNA recognition, but also highlights the possible pitfalls of studying isolated RNA fragments. In the crystal structure, the stem-loop IV that forms a hairpin in solution reorganizes to form a bulged double-helical structure that binds two p65 domains [27].

**Figure 3:**
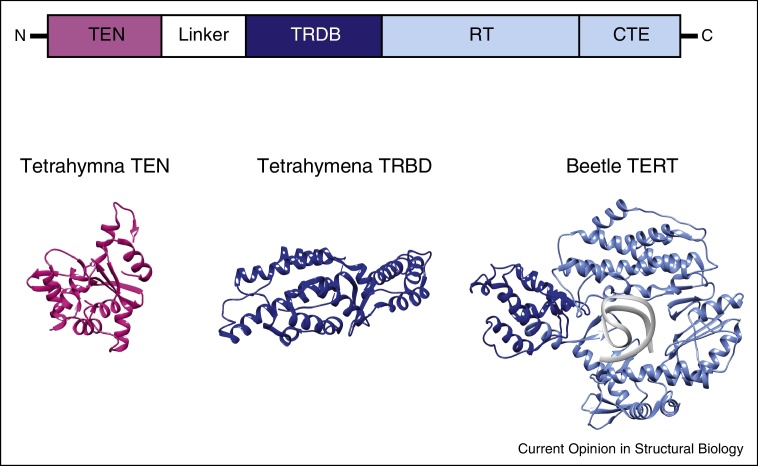
Domain structure of the TERT catalytic subunit. TERT contains a telomerase essential N-terminal (TEN) domain, a flexible linker region, a telomerase RNA-binding (TRBD) domain, a reverse transcriptase (RT) domain and a C-terminal domain (CTE). The TEN domain (pink) binds single-stranded telomeric DNA upstream of the primer-template. The TRBD domain (dark blue) binds the pseudo-knot and the transactivation domain of TER. Crystal structures of isolated domains from Tetrahymena: TEN domain (pdb number: 2B2A) is depicted in pink and TRDB (pdb number: 4R4G) domain in dark blue. The crystal structure of the beetle TERT (dark and light blue) shows a ring-like structure in complex with a RNA-DNA hairpin (pdb number: 3KYL).

Sequence alignments of the TERT genes from different species showed that the telomerase catalytic subunit has a largely conserved domain organization and size (around 1100 amino acids) (reviewed in [32]). The central reverse transcriptase domain (RT) shares significant structural and functional homology with retroviral reverse transcriptase [33]. Mutations of conserved residues in the catalytic core abolish telomerase activity, leading to telomere shortening [10]. The RT domain is flanked by an essential N-terminal extension domain (TEN), which has binding affinity for single-stranded telomeric DNA and also interacts directly with TPP1/TIN2. The TEN domain is linked to the RNA binding domain (TRBD) by an unstructured linker. The TRBD has been shown to interact with the CR4/5 region of TER. C-terminally to the RT is the C-terminal extension CTE (reviewed in [26,32]). The most functionally insightful structural information comes from the crystal structure of the full-length beetle *Tribolium castaneum* TERT in complex with a short DNA–RNA helix [34] (Figure 3). The TERT subunit forms a ring-like structure in which TRBD and CTE come close together in space forming a tunnel, which contains the catalytic pocket [33]. Importantly, the structure reveals the details of DNA substrate binding by the thumb domain in the CTE, and RNA template binding by the finger and palm domains in the RT, thus positioning the 3′ end of the G-overhang at the active site for nucleotide addition (reviewed in [35]). The beetle TERT subunit lacks the TEN domain, but the crystal structure of the isolated *Tetrahymena* TEN domain as well as that of the isolated TRBD have been determined (Figure 3) [36,37].

## Structure of human and *Tetrahymena* telomerase

**Figure 4:**
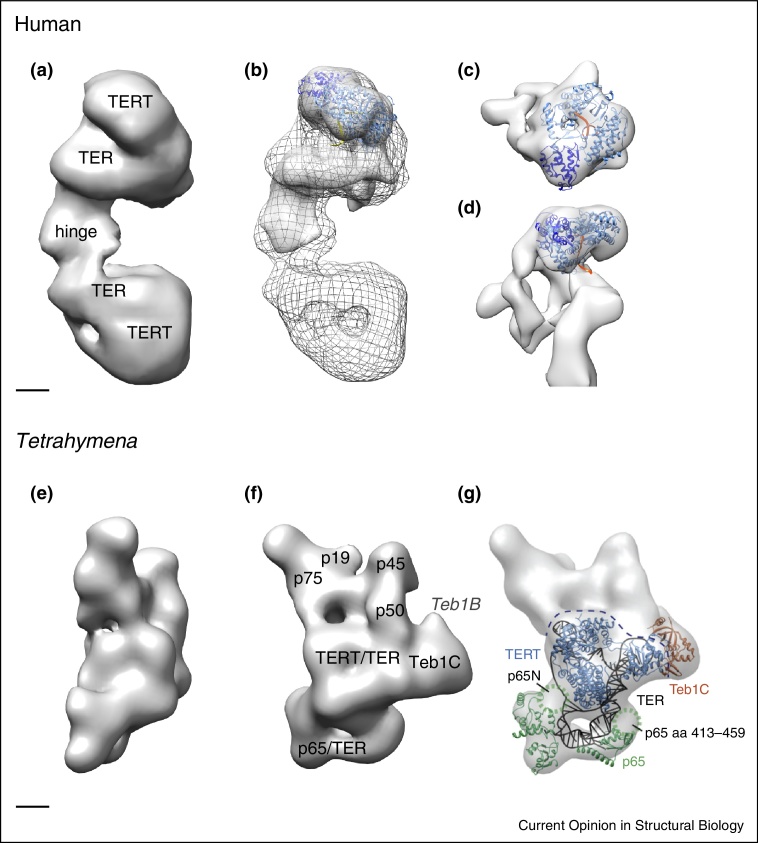
3D structures of human and *Tetrahymena* telomerases. Single particle analyses of full-length human (a–d) and *Tetrahymena* (e–g) holoenzyme complexes in negative stain. **(a)** The 3D reconstruction of the human telomerase dimer at 30 Å resolution showing two monomers connected by a flexible hinge region. **(b)** Independent refinement of one half of the dimer, at a resolution of 23 Å, superimposed with the dimer density (wireframe). **(c and d)** Two views, related by a 90° rotation around the horizontal axis, of the refined monomer together with the docked crystal structure of beetle TERT. TERT is coloured blue, with the TRBD domain in dark blue, and the DNA strand in orange. **(e and f)** Two views of the *Tetrahymena* telomerase. 3D reconstruction at 25 Å resolution. The two views are related by a 90° rotation around the vertical axis. The location of TERT, TER and accessory proteins p75, p19, p45, p50, Teb and p65 are indicated. **(g)** Docking of the atomic structures p65, Teb1C, homology model *Tetrahymena* TERT (blue), and RNA model of TER (black) into the EM density map, reproduced with permission from [12].

The first glimpse at the 3D architecture of active, full-length telomerase has come from two structural determinations: one of the human telomerase [15^••^] and the other *Tetrtahymena thermophila* telomerase [16^••^]. Both structures were determined by single particle electron microscopy (EM) in negative stain, at a resolution of about 25 Å. The two EM reconstructions reveal striking differences in both oligomeric state and subunit composition, whilst preserving a similar TERT domain organization (Figure 4).

To enable the 3D structural determination of this low abundance enzyme, human telomerase was overexpressed in HEK293 cells [15^••^,38]. The affinity-purified holoenzyme was shown to be fully active in telomere repeat addition, and has a molecular weight of 670 kDa, consistent with it being a dimer consisting of two TER (153 kDa) and two TERT (127 kDa) subunits, as previously reported for telomerase isolated from immortal cell lines [39]. Non-quantitative mass spectrometry also identified the presence of two additional proteins, Nop10 (7.7 kDa) and dyskerin (58 kDa), involved in telomerase assembly [40] but dispensable for activity [41^•^]. Electron micrographs of the human telomerase show a bipartite particle consisting of two lobes. Bound G-overhang substrate labelled with colloidal gold was used to demonstrate the presence of two TER subunits per telomerase particle, consistent with previous functional studies [42,15^••^]. Biochemical experiments coupled with mutagenesis of the TERT catalytic pocket [43] provide strong evidence that human telomerase contains two TERT subunits, and functions as a dimer. Single particle analysis combined with maximum likelihood classification was used to obtain the 3D reconstruction of the telomerase dimer at 30 Å resolution. The structure reveals both ‘open’ and ‘closed’ monomer conformations, linked by a flexible hinge domain (Figure 4a and b). The origin of the ‘open’ and ‘closed’ conformations is unknown, but presumably reflects different functional states. A refined reconstruction of the ‘open’ monomer at 23 Å resolution (Figure 4c and d) allowed the unambiguous docking of the crystal structure of the beetle TERT [34,33] into the EM density as they share a very distinct shape and also the characteristic tunnel, locating the position of the catalytic pocked in the EM density (Figure 3). This places the TERT subunits at the periphery, locating the catalytic pockets about 180 Å apart in a telomerase dimer. The remaining EM density is likely to be primarily from TER. Tubular regions with a diameter of 20–30 Å, as expected from RNA double-helices or triple-helices, are present in the EM density and two of these regions are in close proximity of TERT. These are likely to correspond to the pseudo-knot and the CR4/CR5 stem-loop TER regions, implicated by functional and cross-linking experiments in TERT binding [44]. It was suggested that the human telomerase may need to be dimeric to permit the extension of two telomeric ends in parallel, enabling sister chromatids to maintain equal telomere length [15^••^].

In contrast to the human telomerase complex, the affinity-purified telomerase from *Tetrahymena* is monomeric [16^••^] and is known to function as a monomer [13]. It has a MW of about 500 kDa [45] and contains one TERT subunit, a small TER subunit (159 nucleotides versus 451 nucleotides in humans) and five additional ciliate-specific telomerase accessory proteins. The accessory proteins have been implicated in both telomerase assembly and activity [46,14,47]. *In vitro* telomerase activity assays with the reconstituted *Tetrahymena* telomerase complex suggest they aid processivity [16^••^], whereas recombinant telomerase was reported to be processive in the absence of the accessory proteins [13]. The EM analysis involved using individually FLAG-tagged TERT and accessory proteins combined with antigen-binding fragment (Fab) labelling. The telomerase complex had preferred orientations on the EM grid, with the broad side facing the carbon support film and 3D reconstructions were carried out by random conical tilt at a resolution of 25 Å in negative stain. The Fab bound FLAG-tags were used to map the location of p65, p75 and p17 in 2D class averages and more precisely, TERT, Teb1 and p50 in 3D reconstructions. The interpretation of the EM density shows the TERT subunit centrally located surrounded by the accessory proteins (Figure 4e–g). In addition to the assignment of the location of subunits in the EM density, a full atomic model for the catalytic core consisting of the TERT, TER and p65 subunits is presented. The model was obtained by fitting the known NMR and crystal structures of TER and TERT into the EM density map, as well as making use of results from functional studies (Figure 4g) [16^••^]. The overall fit of the TERT subunit into the map and its close proximity to TER is as expected, and shows similar features to the human telomerase structure (Figure 4c and d). However, since it is not possible to distinguish small proteins, or proteins domains from RNA helices and loops in an EM reconstruction at 25 Å resolution, the all atom model presented for the catalytic core is speculative.

## Conclusions

The EM structural analyses of the *Tetrahymena* and human telomerases have provided the first view of the 3D architecture of two fully active holoenzymes. The differences observed raise important questions on the functional requirements of the oligomeric state of the telomerase enzyme and importantly, form the basis for future structural and functional studies. To understand the mechanism of telomere elongation fully, higher resolution structures will be required. Recent advances in data acquisition of cryo EM images should enable the elucidation of the different conformational states during the telomerase reaction cycle, as well as provide detailed information on role of accessory proteins in telomerase complex formation and enzymatic activity. The answer to the puzzle of monomeric versus dimeric telomerase complexes as well as holoenzeme composition may reflect that the mechanism of telomere maintenance has evolved in response to the different replicative requirements of ciliate macronuclear nano chromosomes and vertebrate sister chromatids.

## References and recommended reading

Papers of particular interest, published within the period of review, have been highlighted as:

• of special interest
•• of outstanding interest
